# Hepatitis B virus genotypes A1, A2 and E in Cape Verde: Unequal distribution through the islands and association with human flows

**DOI:** 10.1371/journal.pone.0192595

**Published:** 2018-02-15

**Authors:** Isabel Inês M. de Pina-Araujo, Natalia Spitz, Caroline C. Soares, Christian Niel, Barbara V. Lago, Selma A. Gomes

**Affiliations:** 1 Faculdade de Ciência e Tecnologia, Universidade de Cabo Verde, Praia, Santiago, Cape Verde; 2 Laboratório de Virologia Molecular, Instituto Oswaldo Cruz, FIOCRUZ, Rio de Janeiro, Brazil; 3 Instituto de Tecnologia em Imunobiológicos (Bio-Manguinhos), FIOCRUZ, Rio de Janeiro, Brazil; University of Cincinnati College of Medicine, UNITED STATES

## Abstract

Hepatitis B virus (HBV) diversity has not been previously studied in Cape Verde. The archipelago was discovered in 1460 by Portuguese explorers, who brought African slaves to colonise the islands. In this study, we investigated the HBV characteristics from 183 HBsAg-positive Cape Verdean individuals. Phylogenetic analysis of the pre-S/S region and the full-length genomes revealed 54 isolates with HBV/A1 (57%), 21 with HBV/A2 (22%), 19 with HBV/E (20%), and one with HBV/D (1%). HBV genotypes and subgenotypes were unequally distributed through the islands. In São Vicente, the main northern island, most isolates (84%) belonged to the African-originated HBV/A1, with the remaining isolates belonging to HBV/A2, which is prevalent in Europe. Interestingly, the HBV/A1 isolates from São Vicente were closely related to Brazilian sequences into the Asian-American clade, which suggests the dissemination of common African ancestors through slave trade. In contrast, in Santiago and nearby southern islands, where a recent influx from different populations circulates, a higher diversity of HBV was observed: HBV/A1 (40%); HBV/E (32%); HBV/A2 (28%); and HBV/D (1%). HBV/E is a recent genotype disseminated in Africa that was absent in the era of the slave trade. African and European human flows at different times of the history may explain the HBV diversity in Cape Verde. The possible origin and specifics of each HBV genotype circulating in Cape Verde are discussed.

## Introduction

Hepatitis B virus (HBV) infection remains a major cause of liver disease worldwide. It is estimated that two billion people have been infected with HBV and more than 240 million are chronic carriers [1]. HBV infection is highly endemic in Asia and Africa. Among African countries, the prevalence of HBV surface antigen (HBsAg) varies from 5% to up to 15% [2–4]. Despite this, HBV epidemiology is still poorly documented in most African countries [5].

**Fig 1:**
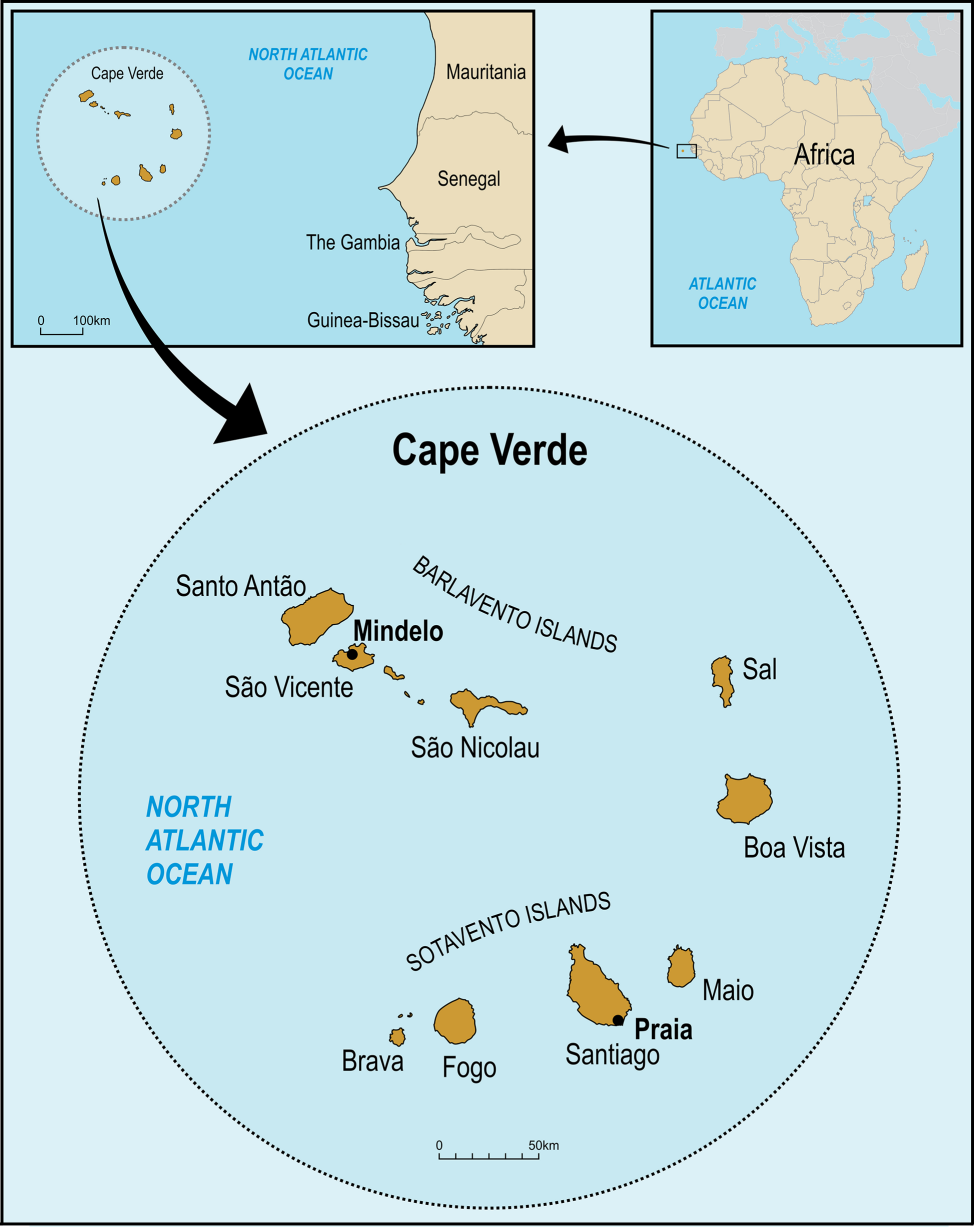
Map of Cape Verde showing the localization of the main islands.

Cape Verde is an archipelago formed by ten islands near the coast of West Africa (Fig 1). These islands were uninhabited until 1460 when they were discovered by Portuguese explorers. Positioned on the great maritime trade routes, the archipelago prospered from the transatlantic slave trade in the 16th century, selling African captives to the Americas and West Indies (Antilles and Central America) [6–8]. The explorers went to Cape Verde without their families and formed liaisons with slave women. This initial admixture of European men, African women and their descendants would become the majority of Cape Verdean population [6].

The first settlement occurred in Santiago, which is the largest island located in southern part of Cape Verde, with Praia being the national capital. The other southern (Sotavento) islands are Brava, Fogo and Maio (Fig 1). São Vicente, Santo Antão and São Nicolau are northern (Barlavento) islands. Mindelo on São Vicente is the second most populated city. Sal and Boa Vista islands, located closer to the African continent, were the later-settled islands [6]. Nowadays, Cape Verde has a population of approximately 500,000 inhabitants, with 70% under 35 years of age. Recent tourism has increased European and American population flows [9] which may contribute to viral diversity in Cape Verde. Local studies showed a frequency of 2.6% of HBsAg among blood donors [10,11].

To date, ten HBV genotypes (HBV/A to HBV/J) have been characterized (for review, see [12]). The genomes of the most recently characterized genotypes, HBV/I [13] and HBV/J [14], are likely HBV/C recombinants. HBV/J has only been isolated from one Japanese patient [14]. HBV genotype classification is based on a sequence divergence of greater than 8% of the entire genome [15,16]. The significant diversity within HBV genotypes A-D and F has led to division into numerous subgenotypes, based on intergroup nucleotide divergences between 4% and 7.5% [17,18]. HBV genotypes and subgenotypes have distinct geographical distributions and may be responsible for differences in the natural history and clinical course of infection [19–22]. In Africa, genotypes A, D and E are predominant. HBV/A and HBV/D are distributed globally, with a high predominance of HBV/A in northwest Europe, North America, South Africa, and Brazil [15,23,24]. HBV/D is predominant in the Euro-Mediterranean area, Portugal, India, Russia and North Africa [19,25]. HBV/E is confined to Africa, being by far the most prevalent in west and central Africa [26].

Genotype A is now classified as HBV/A1, HBV/A2, quasi-subgenotype A3 (QS-A3) and HBV/A4. With exception of HBV/A2, highly prevalent in Europe and North America, HBV/A subgenotypes are of African origin and related to African people. HBV/A3 (recently re-classified as QS-A3) [17,27,28] was originally isolated in Cameroon and has also been identified in Gambia and Mali. Some QS-A3 isolates, previously named as HBV/A4 and HBV/A5, were identified in Mali and Nigeria, respectively. HBV/A4 (previously named HBV/A6) was detected in Afro-Belgian patients, while HBV/A7 (renamed QS-A3) was detected in some individuals from Cameroon [17]. Isolates QS-A3 and HBV/A4 have been identified exclusively in Africa and African descendants. HBV/A1 is highly prevalent in southeast Africa [5,29] and is predominant among South American people of African ancestry, mainly in Brazil [30]. HBV/A1 has also been found in southern Indians [31]. It has been suggested that HBV/A1 was introduced in South America by African slaves, who would have been carriers of HBV/A1 [32]. HBV/A2, the most frequent subgenotype in northwest Europe and the USA, has also being identified in South Africa [26,27,29,31].

Genotype D is the most widespread, with ten subgenotypes, D1-D10, described so far [18,33]. HBV/D4 is dominant in Oceania [24] and Cuba [34] and has also been found in Haiti, Brazil, Morocco, Rwanda, Somalia, Kenya and Ghana.

HBV/E has been identified in Mali, Burkina Faso, Togo, Benin, Nigeria, Cameroon, the Democratic Republic of Congo, and Angola. Genotype E isolates have low genetic variability with unique features, such a second start codon in the pre-S1 region and the rare *ayw4* serotype [5]. Due to its low genetic variability and the fact that this genotype is found exclusively in Africa or African descendants, Mulders and colleagues [35] suggested that the introduction of genotype E into the human population is a recent event that occurred from the mid to the late 19th century, when the slave trade was over. Despite the possible recent introduction of HBV/E as a human pathogen, different clusters of HBV/E have been identified [36,37].

The aim of this study is to associate the phylogenetic data of HBV with historical facts in order to uncover and shed light on the origins and diversity of HBV subgenotypes in Cape Verde. The characteristics and specifics of each HBV genotype circulating in Cape Verde are discussed.

## Materials and methods

### Ethics statement

The Cape Verde National Ethics Committee in Health and Research and the Research Ethic Committee of Oswaldo Cruz Institute, Rio de Janeiro, Brazil approved the study. Serum samples that tested positive for HBsAg were stored at—20°C in local Cape Verdean health units, codified, anonymously handled, and sent in dry ice to Brazil for HBV molecular analysis. The results of the present research were made available to the Cape Verde National Ethics Committee and to local health services where the serum samples were stored.

### Serum samples, viral DNA extraction and PCR amplification

Blood samples were collected between 2010 to 2016 from individuals living in Boa Vista, Fogo, Maio, São Vicente, Santiago and Santo Antão islands. Sera were stored at -20°C in the only two public hospitals, located in Praia (Santiago) and Mindelo (São Vicente), able to perform HBsAg serology of suspected cases. Samples from 183 individuals, who tested positive for HBsAg by ELISA (Monolisa HBsAg ULTRA, Bio-Rad Laboratories, France), were randomly selected.

HBV-DNA was extracted from 200 μl of serum sample with High Pure Viral Nucleic Acid Kit (Roche Diagnostics, Mannheim, Germany) according to the manufacturer’s instructions. HBV-DNA from pre-S/S region was PCR amplified in semi-nested reaction as previously described [38]. The first round of amplification was performed with PS1-P3 oligonucleotide primers. The second round of amplification was performed with sense primer PS1 and a mixture of two antisense primers, S2 and S22 (S1 Table). Serum samples positive for pre-S/S PCR amplification were further subjected to amplification of the whole genome using primers P1 and P2 (S1 Table) as previously described [39].

### Nucleotide sequencing

PCR products from the pre-S/S region and full-length HBV genomes were purified from agarose gel after electrophoresis and directly sequenced (Big Dye Terminator v3.1 Cycle Sequencing kit, Applied Biosystems, Foster City, CA), using HBV internal primers (S1 Table) as described previously [38,40]. Sequencing reactions were analysed on an ABI 3730 automated sequencer (Applied Biosystems). Sequence alignments were performed by the Clustal X programme using reference sequences of all HBV genotypes/subgenotypes, as previously described [41]. Pre-S/S and full-length sequences of each genotype/subgenotype from Cape Verde were further aligned with representative genomes for which complete genome and geographic localisation were available in GenBank, as previously described [32]. Nucleotide alignments were performed by Clustal in MEGA version 6 software. This software was also used to calculate genetic distances and to deduce the amino acids of each genomic region. Phylogenetic trees were constructed by the maximum likelihood method inferred with the PhyML programme [42] using an online web server [43] under the GTR + I + G nucleotide substitution model selected using the jModeltest v.2 programme and the SPR (Subtree Pruning and Regrafting) branch-swapping algorithm for heuristic tree search. The consistency of the tree topology was estimated with an approximate likelihood-ratio test [44] based on a Shimodaira-Hasegawa-like procedure.

### Statistical analysis

Categorical variables were compared using Fisher's exact and Chi-square tests. All statistical analyses were performed using GraphPad software tests in contingency tables. Differences were considered to be statistically significant when the p-value was less than 0.05.

## Results

The demographic profile of the 183 HBsAg-positive subjects studied here is shown in Table 1. Among the individuals whose gender was known, 104 (59%) were men, and 73 (41%) were women. Their ages ranged from five to 74 years with an average of 35.5 years and a median of 34 years. Only 30 (16%) individuals were more than 51 years old. Adults (20–50 years old) comprised approximately 74% of the studied population.

**Table 1:** Sociodemographic characteristics of the HBsAg positive individuals and genotype distribution.

*Variables*	*Samples (n = 183) n (%)*	*HBV-DNA + (n = 126) n (%)*	*Genotype distribution (n = 95)*				
			A1 (n = 54) n (%)	A2 (n = 21) n (%)	E (n = 19) n (%)	D (n = 1) n (%)	*p*
*Sex*							
* Male*	104 (57)	77 (61)	33 (61)	15 (71)	10 (53)	1 (100)	NS
* Female*	73 (40)	46 (37)	19 (35)	6 (29)	9 (47)	-	
* Unknown*	6 (3)	3 (2)	2 (4)	-	-	-	
*Age (years)*							
* 5–19*	11 (6)	8 (6)	5 (9)	2 (10)	1 (5)	-	NS
* 20–30*	63 (34)	46 (37)	19 (35)	11 (52)	7 (37)	-	
* 31–50*	74 (40)	53 (42)	26 (48)	7 (33)	8 (42)	1 (100)	
* > 50*	30 (16)	16 (13)	2 (4)	1 (5)	3 (16)	-	
* Unknown*	5 (3)	3 (2)	2 (4)	-	-	-	
*Educational status*							
* None/incomplete primary*	30 (16)	19 (15)	6 (11)	1 (5)	5 (26)	-	NS
* Complete primary*	60 (33)	45 (36)	16 (30)	11 (52)	8 (42)	1 (100)	
* Lower secondary*	33 (18)	24 (19)	11 (20)	4 (19)	3 (16)	-	
* Upper secondary/tertiary*	22 (12)	14 (11)	6 (11)	1 (5)	2 (11)	-	
* Unkown*	38 (21)	24 (19)	15 (28)	4 (19)	1 (5)	-	
*Residence*: *Islands*^a^							
* Santiago and Southern islands*	100 (55)	70 (56)	21 (39)	15 (71)	17 (89)	1 (100)	<0.0001
* São Vicente/Santo Antão*	78 (43)	52 (41)	32 (59)	6 (29)	-	-	
* Boa Vista (Eastern)*	5 (3)	4 (3)	1 (2)	-	2 (11)	-	
*Hepatitis B*							
* Acute*^b^	7 (4)	5 (4)	3 (6)	-	-	-	NS
* Chronic*	131 (72)	93 (74)	32 (59)	18 (86)	17 (89)	1 (100)	
* Unknown*	45 (25)	28 (22)	19 (35)	3 (14)	2 (11)	-	

^a^The majority of the samples came from Santiago (n = 97) and São Vicente (n = 77). The other islands make up only 5% of the samples. Southern islands: Brava, Fogo and Maio. São Vicente and Santo Antão are Northern islands.

^b^Acute hepatitis cases were defined by the occurrence of two or more clinical symptoms, such as jaundice, nausea, vomiting, fever abdominal pain, associated with HBsAg positivity.

Among the 145 individuals who gave information on their educational status, 90 (62%) never attended school or at most completed their first educational level. Only 22/145 (15%) had attended upper secondary/tertiary studies. Ninety-seven HBsAg-positive samples were from individuals who lived in Santiago, the first main island located in the southern region of the archipelago. Three additional samples were collected in southern islands close to Santiago (Brava, Fogo and Maia). São Vicente, with 77 collected HBsAg-positive samples, is the second most important island located in the northern region of the archipelago. One sample was collected in Santo Antão. Finally, five samples came from Boa Vista, the easternmost island of Cape Verde. HBV-DNA could be detected in 126 of the 183 (69%) serum samples. There were no significant differences in the HBV-DNA positivity in regards to the demographic characteristics of the participants. The classification of HBV sequences into genotypes and subgenotypes was performed after alignment and construction of a phylogenetic tree of all 95 pre-S/S sequences obtained here, in combination with reference strains from all of the genotypes/subgenotypes of HBV. Among these isolates, HBV full-length genomes were successfully PCR-amplified from 19 isolates. Four different genotypes/subgenotypes were found. HBV/A1 was the most prevalent (n = 54, 57%) followed by HBV/A2 (n = 21, 22%) and HBV/E (n = 19, 20%). One HBV/D isolate was also detected (Table 1). As expected, the deduced amino acids of the small S proteins of all HBV/A isolates corresponded to the *adw2* serotype. HBV/E isolates were from *ayw4* serotype and the HBV/D isolate was *ayw2*.

There were no significant differences in the demographic characteristics of the participants regarding the genotyping distribution, with the exception of the geographic residence of the participants. Although HBV/A1 could be detected in the southern and northern islands, a large proportion of HBV/A1, 32/54 (59%), belonged to the São Vicente northern island. Moreover, in São Vicente, HBV/A1 was the most frequent (32/38, 84%) genotype, with a small proportion of HBV/A2 (6/38, 16%). In contrast, most HBV/A2 and HBV/E isolates were found in Santiago and the nearby southern islands (15/21, 71% and 17/19, 89%, respectively). Contributing to genotype diversity, among the 53 genotyped samples in the southern islands, 21 (40%) belonged to subgenotype A1 and one HBV/D isolate was found in Santiago. In Boa Vista (the easternmost island of Cape Verde), among the three genotyped samples, two belonged to HBV/E and one to HBV/A1. This unequal distribution of HBV genotypes in the archipelago was extremely significant (two-sided P value < 0.0001) (Table 1).

**Fig 2:**
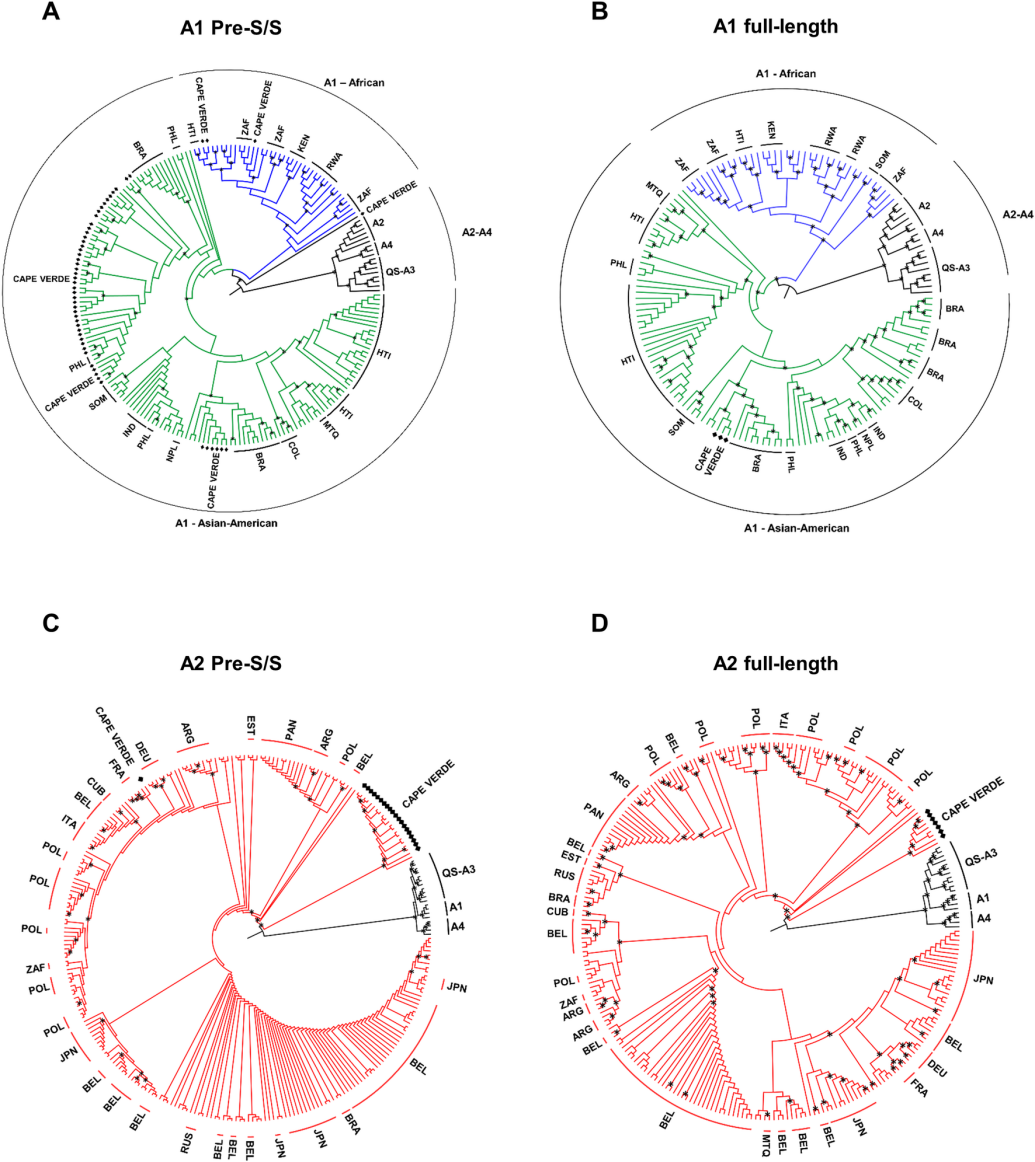
Phylogenetic analysis based on HBV pre-S/S and full-length nucleotide sequences. Phylogenetic trees, performed using the maximum likelihood method, based on HBV pre-S/S region (A,C) and full-length genomes (B,D). One hundred thirty-four HBV/A1 and 231 HBV/A2, whose full-length sequences and geographic origins were available in GenBank, were used for alignment and comparison with 75 HBV sequences from this work (marked with black square). Accession numbers are indicated in S1 File. A,B. Phylogenetic trees of HBV/A1 isolates showing ‘African’ (blue) and ‘Asian-American’ (green) clades. A2, QS-A3 and A4 sequences (black) were included as an outgroup. C,D. Phylogenetic trees of HBV/A2 isolates. All nodes marked with an asterisk showed aLRT support ≥ 0.80. Countries are indicated when two or more neighbor sequences are of common origin.

**Fig 3:**
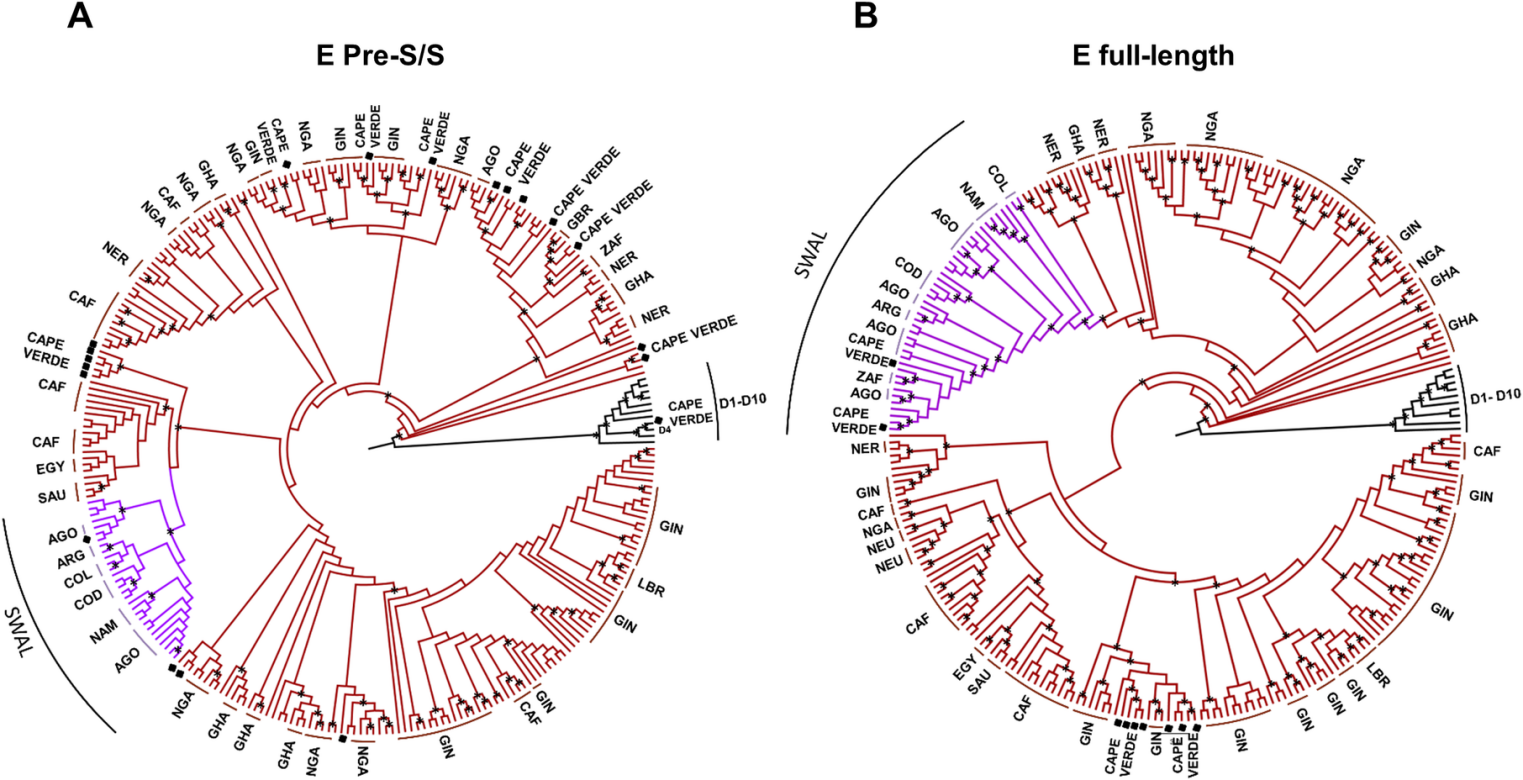
Phylogenetic analysis based on HBV/E pre-S/S and full-length nucleotide sequences. Phylogenetic trees, performed using the maximum likelihood method, based on HBV/E pre-S/S region (A) and full-length genomes (B). Two hundred and fifty HBV/E isolates, whose full-length sequences and geographic origins were available in GenBank, were used for alignment and comparison with 20 HBV sequences from this work (marked with black square). Accession numbers are indicated in S1 File. The Southwest African lineage (SWAL) is shown in purple. All nodes marked with an asterisk showed aLRT support ≥ 0.80. Countries are indicated when two or more neighbor sequences are of common origin.

Fig 2 shows the phylogenetic trees of subgenotypes A1 and A2 found in Cape Verde while Fig 3 represents trees of genotypes D and E. A pair of trees for these genotypes/subgenotypes was constructed based on the pre-S/S nucleotide sequences (Figs 2A, 2C and 3A) and the full-length genome (Figs 2B, 2D and 3B). One hundred thirty-four sequences of HBV/A1, 231 sequences of HBV/A2 and 250 sequences of HBV/E with complete genome and geographic localisation information in GenBank were used for alignment and comparison with sequences from Cape Verde (see supplementary material). The pre-S/S trees were constructed after alignment of the pre-S/S region of the same GenBank sequences with complete genome and geographic region information. Similar phylogenetic trees were obtained for HBV/A genotypes when comparing pre-S/S and full-length trees. Most (50) HBV/A1 isolates, from a total of 54 Cape Verdean HBV/A1 isolates (51 isolates with only pre-S/S sequences and three with complete genome sequences), clustered together with isolates from the ‘Asian-American’ clade (with 0.79 and 0.99 of support for pre-S/S and full-length trees, respectively), represented in green on Fig 2A and 2B. As previously described [31,32], the Asian-American clade grouped sequences from Asian countries (represented here by Bangladesh, India, Japan, Nepal, Philippines, and the United Arab Emirates) and American countries (Brazil, Colombia, Argentina, Martinique and Haiti). Most (5/7) Somalian isolates grouped within this Asian-American clade. The mean distance among full-length sequences from this clade was 1.9 ± 0.1. In contrast, a small number (3/54) of Cape Verdean isolates clustered into the African clade (blue colour) together with the isolates from seven sub-Saharan countries, namely Congo, Kenya, Malawi, Rwanda, Tanzania, Uganda and Zimbabwe, as well as with 16/18 South African isolates (Fig 2A). The two remaining South African isolates clustered into the Asian-American clade; the mean genetic distance within full-length sequences from this clade was 2.3 ± 0.1. One pre-S/S HBV/A1 sequence from Cape Verde (Fig 2A) was outside of both clades. Although a larger proportion (32/54, 58%) of HBV/A1 isolates came from the São Vicente and Santo Antão northern islands, the four sequences grouping outside the Asian-American clade were from Santiago. Overall genetic distance within all full-length HBV/A1 sequences was 2.3 ± 0.1. The three full-length HBV/A1 isolates from Cape Verde clustering in the Asian-American clade were very closely related, with a mean genetic distance of 0.4 ± 0.1. Compared with the Asian-American clade, these three A1 sequences from Cape Verde were more closely related to the 23 Brazilian sequences with a net average distance among them of 0.4 ± 0.1. By pairwise comparison, the distance between them varied from 0.4 to 1.9. The deduced amino acid sequences of all seven HBV open reading frames (polymerase, pre-core/core, pre-S1/pre-S2, S and X protein) for the 188 HBV/A1 isolates (134 reference sequences and 54 isolates from this work) used to construct the phylogenetic trees on Fig 2A and 2B, were compared.

Table 2 highlights seven consensus amino acids of the HBV polymerase of the Cape Verdean A1 isolates. The first four positions, A91, H138, P198, and H269, were shared by the Asian-American clade. These amino acids were found to be largely predominant (88%) in isolates from the Asian-American clade. For all other African countries, with exception of Somalia, typical residues for those positions were I (91), Q (138), S (198), and Y (269). Somalia isolates displayed I91, Q138 and the amino acid consensus of the Asian countries at the other six positions. Cape Verde isolates displayed a unique three amino acid consensus in the polymerase: T356, I601 and S665, with frequencies of 83% for T356 and 100% for the two others. No other unique amino acid consensus of the HBV/A1 isolates of Cape Verde was found in the other open reading frames.

**Table 2:** Frequencies of amino acid residues in HBV/A1 isolates from Cape Verde and comparison with other geographic localities.

Continent	Country/region	Polymerase position (reverse transcriptase domain)						
		91	138	198	269	355 (rt7)	601 (rt253)	665 (rt317)
**Africa**	**Cape Verde**^a^	**A (3/3)**^e^	**H (3/3)**	**P (51/54)**	**H (52/54)**	**T (45/54)**	**I (3/3)**	**S (3/3)**
America	Brazil	**A (23/23)**	**H (23/23)**	**P (20/23)**	**H (23/23)**	D (15/23)	V (22/23)	A (22/23)
	Hispanic ^b^	**A (6/7)**	**H (5/7)**	**P (6/7)**	**H (6/7)**	Variable	V (7/7)	A (7/7)
Asia	Various^c^	**A (16/21)**	**H (17/21)**	**P (19/21)**	**H (21/21)**	A (13/21)	V (21/21)	A (21/21)
Africa	Somalia	I (4/7)	Q (4/7)	**P (5/7)**	**H (6/7)**	A (6/7)	V (7/7)	A (7/7)
	Other countries^d^	I (33/35)	Q (132/35)	S (33/35)	Y (31/35)	V (22/35)	V (35/35)	A (35/35)

^a^Data from this work. Fulll-length genome was sequenced for three samples while pre-S/S region was determined for 54 isolates.

^b^Colombia and Argentine.

^c^Bangladesh, India, Japan, Nepal, Philippines and United Arab Emirates.

^d^Congo, Kenya, Malawi, Rwanda, South Africa, Tanzania, Uganda and Zimbabwe.

^e^Type of amino acid (n/total).

Amino acid positions from Cape Verde and those from other countries with the same amino acid consensus as Cape Verde are highlighted.

Twenty-one out of 22 HBV/A2 pre-S/S sequences from Cape Verde grouped into a single cluster quite separate from the HBV/A2 sequences of the other geographic regions (Fig 2C). The seven full-length genomes of Cape Verde isolates that were successfully sequenced confirmed that the Cape Verde HBV/A2 isolates clustered in a group separated from the others, with 0.99 of support (Fig 2D). Among all 231 isolates analyzed, only one clustered with the HBV/A2 Cape Verdean isolates (Fig 2C and 2D). This sequence was from Poland (accession number GQ477464, see S1 File). The overall mean genetic distance within the 238 HBV/A2 full-length sequences was 1.1 ± 0.1. The genetic distances between the HBV/A2 full-length genomes of Cape Verde and those of the other localities (continental Africa, Asia, Americas or Europe) were similar (approximately 2.2).

Genetic distances of HBV/A2 isolates within countries where they circulate, were determined, based on full-length sequences. Low (0.2–0.4) values were found for Cuba, Brazil, Japan, Belgium, Estonia and Latvia. In other countries (Argentine, Martinique, South Africa, Germany and Italy) they varied from 0.7 to 1.1. The genetic distances between HBV/A2 sequences of Cape Verde were higher (1.8 ± 0.2), as it is the case for Poland (1.7 ± 0.1) and France (1.5 ± 0.1). The highest value (2.2 ± 0.1) was observed in Spain.

Interestingly, the deduced amino acid sequences of the HBV proteins showed that some amino acid consensuses of the Cape Verdean A2 isolates where different from those observed in other regions of the world (Table 3). This is the case for four, 10, and three consensuses in the pre-S1, polymerase, and X protein, respectively. These variations appeared at high frequencies (57–100%) among the isolates from Cape Verde. High rates (78–99%) [37] of other amino acids in these positions were observed for all other HBV/A2 sequences from around the world. In all cases, those differences of frequencies were extremely statistically significant (P < 0.0001).

**Table 3:** Amino acid variability of HBV/A2 isolates.

		Cape Verde		Other countries*	
Region/protein	Position	n/total	%	n/total	%
Pre- S1	41	Q (14/16)	88	P (230/231)	99
	67	F (15/17)	88	L (226/231)	98
	74	V (17/18)	94	I (226/231)	98
	91	V (17/19)	89	I (230/231)	99
Polymerase	138	H (7/7)	100	Q (230/231)	99
	233	S (16/17)	94	P (220/231)	95
	249	V (15/17)	88	A (222/231)	96
	256	C (16/17)	94	Y (220/231)	95
	273	S (17/19)	89	N (230/231)	99
	279	Y (16/19)	84	H (229/231)	99
	291	F (17/19)	89	L (228/231)	99
	315	R (6/7)	86	G (227/231)	98
	323	S (5/7)	71	F (214/231)	93
	345	I (7/7)	100	V (230/231)	99
HBx	129	T (4/7)	57	I (207/231)	90
	132	M (6/7)	86	K (189/231)	82
	133	I (7/7)	100	V (181/231)	78

*See S1 File

Fig 3A and 3B HBV/E show phylogenetic analyses based on the pre-S/S and full-length genomes performed with all 250 complete HBV/E isolates available in GenBank. Nine full-length HBV/E sequences from Cape Verde, as well as ten additional pre-S/S sequences (total = 19), were included. A large majority (n = 225) of the HBV/E sequences from GenBank were from African isolates, since HBV/E originated recently in Africa [26]. The remaining 25 sequences were from Argentina, Belgium, Colombia, Cuba, Mexico, Martinique, United Kingdom, Saudi Arabia and Japan. The genetic distance among all the full-length HBV/E isolates was 2.0 ± 0.1. Different from the HBV/A trees, where similar phylogenetic patterns were observed, the HBV/E trees showed a rather distinct clustering pattern when comparing the pre-S/S region and the full-length genome trees (Fig 3A and 3B). In the pre-S/S analysis, the HBV/E sequences of Cape Verde were scattered in the tree, clustering with sequences from different countries (Angola, Guinea, Nigeria, and United Kingdom). As previously described, the full-length genomes of all isolates from Angola, Namibia and the Democratic Republic of Congo clustered together into a cluster called ‘Southwest African lineage’ (SWAL) [37]. Among the nine full-length Cape Verdean HBV/E isolates, two sequences clustered into SWAL, with support of 0.99. In addition, sporadic sequences isolated in South Africa and Central-South America (Argentina, Colombia, Cuba and Mexico) belonged to the separate SWAL lineage (represented in purple). The other seven full-length genomes from Cape Verde clustered near several sequences from Guinea with support of 0.85 (Fig 3A and 3B).

As previously noted, some variations of amino acids are specific for the SWAL lineage [37]. Table 4 shows that the two sequences from the Cape Verde clustering in the SWAL group displayed I57 in the small S, H177 and L612 in the polymerase gene, and L30 and G36 in the X protein. This was expected because these amino acid residues are consensus inside the SWAL lineage. However, both Cape Verdean isolates belonging to the SWAL lineage displayed G245 in the polymerase, which is the consensus for the African isolates outside SWAL, instead of W245, which is the consensus of isolates inside SWAL. No specific variations were observed for the HBV/E belonging to Cape Verde. Finally, the only HBV/D isolate was more closely related to the HBV/D4 subgenotype (Fig 3A).

**Table 4:** Amino acid variability of HBV/E isolates in African Countries.

		Amino acid (n/total)			
Protein	Position	Inside SWAL^a^		Outside SWAL	
		Cape Verde	Other African countries^b^	Cape Verde	Other African countries^c^
Small S	57	I (2/2)	I (30/31)	T (6/7)	T (181/194)
Polymerase	177	H (2/2)	H (24/31)	Q (6/7)	Q (193/194)
	245	G (2/2)	W (22/31)	G (3/7); W (4/7)	G (172/194)
	612 (rt 267)	L (2/2)	L (27/31)	M (7/7)	M (162/194)
X protein	30	L (2/2)	L (28/31)	V (7/7)	V (184/194)
	36	G (2/2)	G(26/31)	D (7/7)	D (165/194)

^a^Southwest African lineage

^b^18/18 isolates from Angola, 6/6 Namibia, 4/4 Democratic Republic of Congo, 2/2 South Africa, and 1/2 Cameroon

^c^1/2 Cameroon, 28/28 Central African Republic, 2/2 Egypt, 1/1 Ethiopia, 17/17 Ghana, 69/69 Guinea, 2/2 Ivory Coast, 6/6 Liberia, 1/1 Madagascar, 17/17 Niger, 47/47 Nigeria, 1/1 Somalia, and 2/2 Sudan.

## Discussion

Two main HBV genotypes, A and E, were observed in Cape Verde. Both are frequently found in Africa, with one or the other being highly prevalent in most African countries. Despite the high variability of African HBV/A, only two subgenotypes, HBV/A1 and HBV/A2, were detected in Cape Verde. HBV/A1 was the most frequently detected subgenotype in Cape Verde. It is also the major African genotype spread among Afro descendants outside of Africa. It is likely that HBV/A1 was spread by the slave trade, which exported African slaves to Asia in the 17th century as a result of Arab or Portuguese trade and to Latin America in the 16th to 19th centuries through the trans-Atlantic slave trade [31].

By phylogenetic analysis, we showed that most HBV/A1 isolates from Cape Verde clustered into the Asian-American clade and these isolates were very closely related to the HBV/A1 isolates from Brazil (Fig 2A and 2B). This identity of the HBV genomes may be explained by the spread of HBV/A1 during the Atlantic slave trade. In fact, it is known that most of the slaves brought to Cape Verde by the Portugueses were en route to Brazil and the West Indies (Antilles and Central America) [6–8]. The question of the location of the forcible capture of these Africans that spread HBV/A1 remains unanswered. Tracing the routes of African slavery is difficult, as the Portuguese mixed different ethnic groups captured in different African regions, exporting them inside and outside of Africa. Our results and other studies [31,32,45,46] show that inside Africa, other than Cape Verde, only Somalia has a significant number of isolates clustering into the HBV/A1 Asian-American clade. Moreover, some other Somalian isolates cluster into the African clade (Fig 2A and 2B). Bantu people, disseminated in Africa at the time of slavery, seem to be the link between Somalia, Asia, Cape Verde and Brazil. Bantu people in Somalia may be the origin of the HBV/A1 that differentiated into the two clades (African and Asian-American) since it was dispersed through Asian and American countries. Other possibility for the geographic origin of HBV/A1 is Mozambique, from which Africans were captured for slavery between 1837 and 1856, circumventing the laws that banned the transatlantic slave trade [32,47]. To date, no HBV full-length sequences from this locality have been published. Other previous studies corroborate that the dispersion of HBV/A1 through continents was via Somalia and/or Bantu people during the Atlantic slave trade [31,45,46,48]. However, it is not possible to rule out the hypothesis that HBV/A1 originated from western African countries (with Angola and Congo as the major sources of slaves to Brazil) and that HBV/A has now been replaced by HBV/E.

HBV/A2 was the second most prevalent subgenotype in Cape Verde, with 21/95 (22%) isolates. HBV/A2 is frequently detected in northwest Europe and the USA, and has been isolated in South Africa [26,27,29,31]. Several studies have suggested that the dispersion of HBV/A2 should be more recent than that of HBV/A1 [48,49]. The evolutionary pattern of HBV/A2 suggests an exponential growth of infections between 1970 and the mid-1990s [49]. The spread of HBV/A2 seems to be linked to sexual transmission, since HBV/A2 is more prevalent in sexual behavioural risk groups, such as men who have sex with men [50–53]. Although it is possible that HBV/A2 was disseminated at the time of slavery, as suggested previously [46], the origin of the currently circulating HBV/A2 seems to be more recent than that of HBV/A1, and would have occurred during the first decades of the 20th century, as described above. Indeed, we observed that the isolates from Cape Verde did not cluster with the isolates from South Africa (Fig 2C and 2D), suggesting that the introduction of HBV/A2 into Cape Verde had not occurred via South Africa. HBV/A2 is highly prevalent in Europe, but it is unlikely that HBV/A2 was introduced into Cape Verde by the Portuguese explorers during colonisation. It is known that Portuguese settlers had a low contribution in the formation of the Cape Verdean people, as shown by historical facts and by a mitochondrial DNA study [6]. Moreover, there is a consensus that HBV/A2 is more recent than HBV/A1 [46]. Indeed, we found that the genetic distances within all of the isolates of HBV/A2 were much lower (1.1 ± 0.1) than that within HBV/A1 (2.3 ± 0.1), in agreement with studies demonstrating that the HBV/A2 subgenotype is more recent than that of HBV/A1 [48,49]. It is probable that HBV/A2 introduction to Cape Verde occurred during or after the exponential growth of HBV/A2 in Europe. Corroborating a recent European origin of the HBV/A2 isolates in Cape Verde, the only full-length sequence that clusters with the Cape Verdean HBV/A2 isolates was a sequence from Poland. The genetic distances within Cape Verde isolates (1.8 ± 0.1) were similar to those observed within some European countries such as Poland (1.7± 0.1) and France (1.5 ± 0.1), thereby suggesting a similar time of infection/differentiation in Cape Verde, as in some European countries.

Several amino acid consensuses of the Cape Verdean HBV/A1 and HBV/ A2 isolates were different from those observed in other regions of the world. Two of these variations, namely pre-S1 74V and 91V, observed among the HBV/A2 isolates from Cape Verde, were present in almost all 151 HBV/A1, QSA3, HBV/A4 isolates used for sequence alignment. This raises the hypothesis that HBV/A2 isolates from Cape Verde to be intersubgenotypic recombinants. However, no evidence of recombination was observed for any Cape Verdean sequence when analyzed by RDP4 and Simplot recombination programs. Other two variations in HBV/A2, namely 132M and 133I in X protein, correspond to the well studied double mutation 1762T-1764A in the basal core promoter, associated with the anti-HBe phenotype. This double mutation has been observed in different genotypes of HBV. However, its frequency was much higher in HBV/A2 from Cape Verde than from other countries.

While HBV/A predominates in eastern and south eastern Africa, HBV/E is the most frequent genotype in a large area of central and western Africa [26,49]. Out of Africa, the HBV/E isolates have sporadically been found within the Americas, indicating that this genotype was introduced into the general African population after the end of the trans-Atlantic slave trade [26], likely within the last 130 years [54]. Distinct from most genotypes, HBV/E has a low degree of genetic diversity, and HBV/E isolates are classified into a single monophyletic group [35]. In this study, HBV/E was detected only in the southern and Boa Vista islands, with an overall frequency of 20% (19/95 isolates). The genetic distance within all HBV/E isolates (2.0 ± 0.1) was similar to that within a single subgenotype (HBV/A1; 2.3 ± 0.1). This relative low degree of variability is probably due to the more recent origin of HBV/E. However, pre-S/S and full-length phylogenetic analyses showed that HBV/E isolates from Cape Verde did not cluster together (Fig 3A and 3B), thereby suggesting different origins/introductions of HBV/E circulating in Cape Verde.

In contrast to HBV/A1 and HBV/A2, no specific consensus of amino acids was observed among the HBV/E isolates circulating in Cape Verde, indicating that HBV/E was more recently introduced in Cape Verde than HBV/A1 and HBV/A2. The lower prevalence of HBV/E in relation to HBV/A1 indicates that there has not been, at least so far, any explosion of HBV/E in Cape Verde, as is supposed to have occurred in western Africa [26].

In this study, we observed an unequal distribution of the HBV genotypes throughout the Cape Verde islands. São Vicente and the northern islands showed a high prevalence of HBV/A1 from the Asian-American clade, a low prevalence of HBV/A2 and the absence of the recent HBV/E genotype. This distribution is as expected for the time of the discovery of the archipelago: a high frequency of the HBV/A1, all of them belonging to the Asian-American clade associated with the slave traffic, and a small percentage of HBV/A2 that corresponds to the European genotype. In contrast, Santiago and the southern islands displayed a mixture of ancient and recent HBV genotypes (HBV/A1/A2/E and D). Furthermore, Santiago displayed HBV/A1 sequences grouping inside and outside of the Asian-American clade. HBV genotype prevalence in the southern islands may reflect the ancient and modern influx of human population in Cape Verde.

In conclusion, the diversity of HBV genotypes observed in Cape Verde seems to be linked to the ancient historical relationship and the modern relationship of Cape Verde with African and European countries.

## Supporting information

S1 File Full-length genome sequences used to construct phylogenetic trees (Figs 2 and 3). Sequences are identified by their GenBank accession numbers and countries of origin. The following criteria were used for inclusion in the phylogenetic studies: non-recombinant human isolates with known country of origin whose nucleotide sequences have been totally determined and did not show any insertion.
(DOCX)

S1 Table Primers used for PCR amplification and sequencing. (DOCX)
