# Supplementary material for: Hepatitis B virus genotypes A1, A2 and E in Cape Verde: Unequal distribution through the islands and association with human flows
Source: PLoS One. 2018 Feb 15;13(2):e0192595. doi: 10.1371/journal.pone.0192595 (PMC5813952; doi:10.1371/journal.pone.0192595)
Supplement: S1 File — Sequences are identified by their GenBank accession numbers and countries of origin. The following criteria were used for inclusion in the phylogenetic studies: non-recombinant human isolates with known country of origin whose nucleotide sequences have been totally determined and did not show any insertion. (DOCX) [file pone.0192595.s001.docx]

**Supplementary material**

**S1 File. Full-length genome sequences used to construct phylogenetic trees (Figs 2 and 3).** Sequences are identified by their GenBank accession numbers and countries of origin. The following criteria were used for inclusion in the phylogenetic studies: non-recombinant human isolates with known country of origin whose nucleotide sequences have been totally determined and did not show any insertion.

**1. HBV/A1 trees (Figs 2A and 2B) were constructed with 134 HBV/A1 reference isolates, 54 HBV/A1 sequences from Cape Verde (3 full-length genomes, 51 pre-S/S sequences) and 17 outgroup isolates.**

ARE: United Arab Emirates (n= 1). DQ020003

ARG: Argentina (n= 3). AF043560, EU185789, EU366129

BGD: Bangladesh (n=2). AB116084, AB116085

BRA: Brazil (n= 23). KJ854707, KJ854706, KJ854705, KJ854704, KJ854703, KJ854702, KJ854701, KJ854700, KJ854699, KJ854698, KJ854697, KJ854696, KJ854695, KJ854693, KJ854694, KJ854692, KJ854691, KJ854690, KJ854689, KJ854688, KJ854687, KJ854686, KJ854685

COG: Democratic Republic of the Congo (n= 1). DQ020002

COL: Colombia (n= 4). JQ023660, JQ023661, JQ023662, JQ023663

CPV: Cape Verde (n= 54). MF772351, MF772352, MF772353, MF772363, MF772364, MF772365, MF772366, MF772367, MF772368, MF772369, MF772370, MF772371, MF772372, MF772373, MF772374, MF772375, MF772376, MF772377, MF772378, MF772379, MF772380, MF772381, MF772382, MF772383, MF772384, MF772385, MF772386, MF772387, MF772388, MF772389, MF772390, MF772391, MF772392, MF772393, MF772394, MF772395, MF772396, MF772397, MF772398, MF772399, MF772400, MF772401, MF772402, MF772403, MF772404, MF772405, MF772406, MF772407, MF772408, MF772409, MF772410, MF772411, MF772412, MF772413

FRA: France (n=1). AJ309369

HTI: Haiti (n= 34). FJ692557, FJ692558, FJ692559, FJ692560, FJ692561, FJ692562, FJ692563, FJ692564, FJ692565, FJ692566, FJ692567, FJ692568, FJ692570, FJ692571, FJ692572, FJ692573, FJ692574, FJ692575, FJ692576, FJ692577, FJ692578, FJ692579, FJ692580, FJ692581, FJ692582, FJ692583, FJ692584, FJ692585, FJ692586, FJ692587, FJ692589, FJ692590, FJ692591, FJ692592

IND: India (n= 7). AB116086, AB116087, AY161140, AY373429, DQ315784, DQ315785, DQ315786

JPN: Japan (n=2). AB453986, AB453988

KEN: Kenya (n= 4). JX154579, JX154580, JX154581, JX154582

MTQ: Martinique (n= 6). HE974362, HE974363, HE974365, HE974370, HE974375, HE974381

MWI: Malawi (n=2). AB076678; AB076679

NPL: Nepal (n= 2). AB116088, AB116089

PHL: Philippines (n= 7). AB116091, AB116092, AB116093, AB116094, AY934774, EU410082, M57663

RWA: Rwanda (n= 7). FM199974, FM199976, FM199977, FM199978, FM199979, FM199980, FM199981

SOM: Somalia (n= 7). AY934765, AY934766, AY934767, AY934768, AY934769, AY934770, AY934771

TZA: United Republic of Tanzania (n= 1). AY934773

UGA: Uganda (n= 1). AY934772

ZAF: South Africa (n= 18). AF297623, AY233274, AY233275, AY233276, AY233277, AY233278, AY233279, AY233281, AY233282, AY233283, AY233284, AY233285

ZWE: Zimbabwe (n= 1). HM535205

Outgroup Sequences

A2 (n= 6). AB453979, AY233286, HE576988, KJ854710, KJ854709, KJ854708

QS-A3 (former A3) (n= 5). AY934764, FN545825, GQ161813, AM180623, KP234052

QS-A3 (former A5) (n= 3). FJ692554, FJ692556, FJ692610

A4 (former A6) (n=3). GQ331046, GQ331047, GQ331048

**2. HBV/A2 trees (Figs 2C and 2D) were constructed with 231 HBV/A2 reference sequences, 21 HBV/A2 sequences from Cape Verde (7 full-length, 14 pre-S/S) and 6 outgroup isolates.**

ARG: Argentina (n=14). AF043580, KJ843166, KJ843172, KJ843173, KJ843182, KJ843184, KJ843186, KJ843188, KJ843192, KJ843214, KJ843215, KJ843216, KJ843217, KJ843218

BRA: Brazil (n= 3). KJ854708, KJ854709, KJ854710

CAF: Central African Republic (n= 72). EU859898, EU859899, EU859900, EU859901, EU859902, EU859903, EU859904, EU859905, EU859906, EU859907, EU859908, EU859909, EU859910, EU859911, EU859912, EU859913, EU859914, EU859915, EU859916, EU859917, EU859918, EU859919, EU859920, EU859921, EU859922, EU859923, EU859924, EU859925, EU859926, EU859927, EU859928, EU859929, EU859930, EU859931, EU859932, EU859933, EU859934, EU859935, EU859936, EU859937, EU859938, EU859939, EU859940, EU859941, EU859942, EU859943, EU859944, EU859945, EU859946, EU859947, EU859948, EU859949, EU859950, EU859951, EU859953, EU859954, EU859955, EU859956, FJ349224, GU563546, GU563550, GU563551, GU563553, GU563554, GU563555, GU563557, GU563558, GU563562, KP234051, KP274925, KP274927, KP274928

CPV: Cape Verde (n=21). MF772344, MF772345, MF772346, MF772347, MF772348, MF772349, MF772350, MF772415, MF772416, MF772417, MF772418, MF772419, MF772420, MF772421, MF772422, MF772423, MF772424, MF772425, MF772426, MF772427, MF772428

CUB: Cuba (n=3). KM606742, KM606746, KM606749

DEU: Germany (n=8). AY738139, AY738140, AY738141, AY738142, AY738143, DQ788725, EF208113, EU086721

ESP: Spain (n=3). AJ627226, AJ627227, AJ627228

EST: Estonia (n=4). EU594383, EU594384, EU594385, EU594386

FRA: France (n= 4). AJ309369, AJ344115, HE576988, HE576989

ITA: Italy (n=5). DQ298161, DQ298162, DQ298163, DQ298164, DQ298165

JPN: Japan (n= 33). AB362931, AB697487, AB697488, AB697489, AB697491, AB697492, AB697493, AB697495, AB697496, AB697497, AB697498, AB697499, AB697501, AB697503, AB697504, AB697505, AB697506, AB697507, AB697508, AB697509, AB697511, AB697512, AB775198, AB775199, AB775200, AB775201, AB937792, AB937793, AB937794, AB937797, AB937798, LC074724, LC150336

LVA: Latvia (n= 2). EU594387, EU594388

MTQ: Martinique (n= 6). HE974364, HE974367, HE974371, HE974374, HE974376, HE974383

NLD: Netherlands (n= 2). JX310722, JX310723

PAN: Panama (n= 15). KP718088, KP718089, KP718090, KP718091, KP718092, KP718093, KP718094, KP718095, KP718096, KP718097, KP718098, KP718099, KP718100, KP718101, KP718102

POL: Poland (n= 45). GQ477460, GQ477461, GQ477462, GQ477463, GQ477464, GQ477465, GQ477466, GQ477467, GQ477468, GQ477469, GQ477470, GQ477471, GQ477472, GQ477473, GQ477474, GQ477475, GQ477476, GQ477477, GQ477478, GQ477479, GQ477480, GQ477481, GQ477482, GQ477483, GQ477484, GQ477485, GQ477486, GQ477487, GQ477488, GQ477489, GQ477490, GQ477491, GQ477492, GQ477493, GQ477494, GQ477495, GQ477496, GQ477497, GQ477498, GQ477499, GQ477500, GQ477501, GQ477502, GQ477503, GQ477504

RUS: Russian Federation (n=7). EU594389, EU594390, EU594391, EU594392, EU594393, EU594394, EU594395

URY: Uruguay (n= 1). KJ586809

VEN: Venezuela (n= 1). KP995108

ZAF: South Africa (n= 3). GQ184323, GQ184324, JX507080

Outgroup sequences

A1 (n= 4). AY373429, AY903452, AY934765, JN182318

QS-A3 (former A3) (n=7). AY934764, KP234052, FN545825, GQ161813, AB194951, AY934764, FN545833

QS-A3 (former A5) (n= 4). FJ692554, FJ692610, FJ692556, FJ692613

A4 (former A6) (n= 4). GQ331046, GQ331047, GQ331048, EU859952

**3. HBV/E trees (Figs 3A and 3B) were constructed with 250 HBV/E reference isolates, 20 sequences from Cape Verde (9 full-length, 10 pre-S/S and 1 pre-S/S from HBV/D) and 10 outgroup isolates.**

AGO: Angola (n=18). DQ060822, DQ060823, KF849713, KF849714, KF849715, KF849716, KF849717, KF849718, KF849719, KF849720, KF849721, KF849722, KF849723, KF849724, KF849725, KF849726, KF849727, KF849728

ARG: Argentina (n= 2). JQ000008, JQ000009

BEL: Belgium (n= 7). AY935700, FJ349226, FJ349227, FJ349237, FJ349238, FJ349239, FJ349240

CAF: Central African Republic (n= 28). AM494689, AM494690, AM494691, AM494692, AM494693, AM494694, AM494695, AM494696, AM494697, AM494698, AM494699, AM494700, AM494701, AM494702, AM494703, AM494704, AM494705, AM494706, AM494707, AM494708, AM494709, AM494710, AM494711, AM494712, AM494713, AM494714, AM494715, AM494717

CIV: Cote d'Ivoire (n= 2). AB091255, AB091256

CMR: Cameroon (n= 2). AB194947, AB194948

COD: Democratic Republic of the Congo (n= 4). AY738144, AY738145, AY738146, AY738147

COL: Colombia (n= 2). JQ023664, JQ023665

CPV: Cape Verde (HBV/D) (n=1). MF772414

CPV: Cape Verde (HBV/E) (n= 19). MF772354, MF772355, MF772356, MF772357, MF772358, MF772359, MF772360, MF772361, MF772362, MF772429, MF772430, MF772431, MF772432, MF772433, MF772434, MF772435, MF772436, MF772437, MF772438

CUB: Cuba (n= 3). KM606738, KM606739, KM606743

EGY: Egypt (n= 2). KU736891, KU736892

ETH: Ethiopia (n= 1). KU736893

GBR: United Kingdom (n= 3). AB219529, AB219533, AB219534

GHA: Ghana (n= 17). AB205129, AB205188, AB205189, AB205190, AB205191, AB205192, AB106564, EU239217, EU239218, EU239219, EU239220, EU239221, EU239222, EU239223, EU239224, EU239225, EU239226

GIN: Guinea (n= 69). GQ161755, GQ161757, GQ161758, GQ161759, GQ161760, GQ161761, GQ161762, GQ161763, GQ161764, GQ161765, GQ161766, GQ161768, GQ161769, GQ161770, GQ161771, GQ161772, GQ161773, GQ161774, GQ161776, GQ161777, GQ161779, GQ161780, GQ161781, GQ161782, GQ161783, GQ161784, GQ161785, GQ161786, GQ161787, GQ161789, GQ161790, GQ161791, GQ161792, GQ161793, GQ161794, GQ161795, GQ161796, GQ161797, GQ161798, GQ161799, GQ161800, GQ161801, GQ161803, GQ161804, GQ161807, GQ161808, GQ161809, GQ161810, GQ161811, GQ161814, GQ161815, GQ161816, GQ161817, GQ161819, GQ161820, GQ161823, GQ161824, GQ161825, GQ161826, GQ161827, GQ161828, GQ161829, GQ161831, GQ161832, GQ161833, GQ161834, GQ161835, GQ161836, KX186584

JPN: Japan (n= 1). AP007262

LBR: Liberia (n= 6). KU736894, KU736895, KU736896, KU736897, KU736898, KU736899

MDG: Madagascar (n= 1). DQ060830

MEX: Mexico (n= 1). KT192626

MTQ: Martinique (n= 2). HE974380, HE974384

NAM: Namibia (n= 6). DQ060824, DQ060825, DQ060826, DQ060827, DQ060828, DQ060829

NER: Niger (n= 17). FN594748, FN594749, FN594750, FN594751, FN594752, FN594753, FN594754, FN594755, FN594756, FN594758, FN594759, FN594760, FN594762, FN594763, FN594764, FN594765, FN594766

NGA: Nigeria (n= 47). HM363565, HM363566, HM363567, HM363568, HM363569, HM363570, HM363571, HM363572, HM363573, HM363574, HM363575, HM363576, HM363577, HM363578, HM363579, HM363580, HM363581, HM363582, HM363583, HM363584, HM363585, HM363586, HM363587, HM363588, HM363589, HM363590, HM363591, HM363592, HM363593, HM363594, HM363595, HM363596, HM363597, HM363598, HM363599, HM363600, HM363601, HM363602, HM363603, HM363604, HM363605, HM363606, HM363607, HM363608, HM363610, HM363611, AB915175

SAL: Saudi Arabia (n= 4). AB915175, AB915176, AB915178, AB915180

SDN: Sudan (n= 2). KF170741, KF170742

SOM: Sudan (n= 1). KU736900

ZAF: South Africa (n= 2). KF922438, KF922439

Outgroup sequences

D1 (n=1). KM606753

D2 (n=1). KR905424

D3 (n=1). HE815465

D4 (n=1). HE974382

D5 (n=1). KP322603

D6 (n=1). KF170740

D7 (n=1). KM606741

D8 (n=1). FN594769

D9 (n=1). JN664942

D10 (n=1). KX357622
