# Supplementary material for: Hepatitis B virus genotypes A1, A2 and E in Cape Verde: Unequal distribution through the islands and association with human flows
Source: PLoS One. 2018 Feb 15;13(2):e0192595. doi: 10.1371/journal.pone.0192595 (PMC5813952; doi:10.1371/journal.pone.0192595)
Supplement: S1 Table — (DOCX) [file pone.0192595.s002.docx]

| Primer | Sense | Sequence 5´- 3´ | Genome Position |
| --- | --- | --- | --- |
|  |  |  |  |
| PCR/Sequencing |  |  |  |
| P1 | S | CCggAAAgCTTgAgCTCTTCTTTTTCACCTCTgCCTAATCA | 1821-1841 |
| P2 | A | CCggAAAgCTTgAgCTCTTCAAAAAgTTgCATggTgCTgg | 1806-1825 |
| PS1 | S | CCATATTCTTgggAACAAgA | 2826-2845 |
| P3 | A | AAAGCCCAAAAGACCCACAA | 1000-1019 |
| S2 | A | gggTTTAAATgTATACCCAAAgA | 819-841 |
| S22^a^ | A | gTATTTAAATggATACCCACAgA | 819-841 |
| Sequencing |  |  |  |
| PS8 | A | TTCCTgAACTggAgCCACCA | 63-82 |
| S1 | S | CTTCTCgAggACTggggACC | 124-143 |
| PS2 | A | ggTCCCCAgTCCTCgAgAAg | 124-143 |
| S4 | S | TgCTgCTATgCCTCATCTTCT | 416-436 |
| SR | A | CgAACCACTgAACAAATggC | 685-704 |
| S18 | S | GGATGATGTGGTATTGGGGGCCA | 743-765 |
| X1 | S | ACCTCCTTTCCATggCTgCT | 1363-1382 |
| X5 | S | ACTCTTggACTCBCAgCAATg | 1662-1682 |
| C1 | S | CTgTggAgTTACTCTCgTTTTTgC | 1935-1958 |
| C8 | A | GAGGGAGTTCTTCTTCTAGG | 2371-2391 |
| P01 | S | ggACTCATAAggTggggAA | 2470-2488 |
| PS4 | S | ACACTCATCCTCAggCCATgCAgTg | 3194-3218 |
|  |  |  |  |

**Supplementary material**

**S1 Table. Primers used for PCR amplification and sequencing**

^a^ Primer S22 was used only for PCR amplification. Numeration considering HBV/A of 3221 bp
